# Foliar plasticity related to gradients of heat and drought stress across crown orientations in three Mediterranean *Quercus* species

**DOI:** 10.1371/journal.pone.0224462

**Published:** 2019-10-28

**Authors:** Sonia Mediavilla, Ignacio Martín, Josefa Babiano, Alfonso Escudero

**Affiliations:** 1 Área de Ecología, Facultad de Biología, Universidad de Salamanca, Campus Miguel de Unamuno, Salamanca, Spain; 2 Dpto. de Botánica y Fisiología Vegetal, Facultad de Biología, Universidad de Salamanca, Campus Miguel de Unamuno, Salamanca, Spain; University of British Columbia, CANADA

## Abstract

Studies on plasticity at the level of a single individual plant provide indispensable information to predict leaf responses to climate change, because they allow better identification of the environmental factors that determine differences in leaf traits in the absence of genetic differences. Most of these studies have focused on the responses of leaf traits to variations in the light environment along vertical gradients, thus paying less attention to possible differences in the intensity of water stress among canopy orientations. In this paper, we analyzed the differences in leaf traits traditionally associated with changes in the intensity of water stress between east and west crown orientations in three *Quercus* species. The leaves facing west experienced similar solar radiation levels but higher maximum temperatures and lower daily minimum water potentials than those of the east orientation. In response to these differences, the leaves of the west orientation showed smaller size and less chlorophyll concentration, higher percentage of palisade tissue and higher density of stomata and trichomes. These responses would confirm the role of such traits in the tolerance to water stress and control of water losses by transpiration. For all traits, the species with the longest leaf life span exhibited the greatest plasticity between orientations. By contrast, no differences between canopy positions were observed for leaf thickness, leaf mass per unit area and venation patterns.

## Introduction

It is predicted that new climatic conditions will affect the patterns of distribution and species composition of forest ecosystems, which will depend on the ability of each species to respond and adapt to the new conditions. Since plant traits differ more between species than at the intraspecific level [1, 2], research has mainly focused on the analysis of changes in leaf morphology and the physiology of different species through environmental gradients, in order to predict which species will be favored in the new climatic scenario [3, 4]. However, in recent years, many researchers have proposed that predicting changes in the distribution of species and the composition of plant communities requires knowledge regarding the phenotypic plasticity exhibited by different species in response to environmental factors [5, 6]. Indeed, given that phenotypic plasticity allows different traits under varying conditions to appear and to attenuate the effects of stress factors, high plasticity can contribute to improve the performance of a plant species and influence its "fitness" and competitive ability [7, 8].

However, the issue of phenotypic plasticity is complex. Intraspecific variability can be studied at three levels: among populations, among individuals of the same population and within a single individual. It has been postulated that the association among leaf traits may vary depending on the scale of the comparison [9], which makes it necessary to consider trait variations at the different scales. The study of differences in leaf traits between populations allows responses to a wider range of environmental conditions to be analyzed. Nevertheless, this approach has the disadvantage that, among different locations, there are many environmental factors that may vary, and it is not always easy to identify the specific factor to which the leaf traits respond. Although on a smaller scale, similar environmental changes may occur between individuals of a population. In addition, with the exception of studies made with clonal populations, both approaches do not allow the separation of the phenotypic and genotypic components of the responses observed. By contrast, since all the leaves of one individual plant share the same genotype, comparisons between them can help to understand the responses to different stress factors in diverse positions of the canopy in the absence of genetic differences. Also, since the environmental variability among leaves within a canopy should be much smaller than that among individuals of a population and among populations, the responses to specific environmental factors may be more precisely identified. Consequently, interest in the plasticity of leaf traits within the canopy profile of trees is currently on the increase [10–12].

Most of the studies on plasticity at the level of a single individual have been based on the analysis of differences in leaf traits in response to variations in the light environment, focusing primarily on the differences between sun leaves (occupying the highest positions) and shade leaves (in the lowest parts of the canopy). Thus, changes in leaf size, thickness, mass per unit area, stomatal density and venation density have been described in numerous species in response to changes in light levels between extreme positions in the canopy [7, 10, 12, 13]. In dense forests, with tall specimens and a high leaf area index, the greatest environmental heterogeneity at the canopy level is probably associated with variations in the amount of light received by the leaves along a vertical gradient. However, in more open forests, where the crowns are not shaded by the surrounding vegetation, other environmental factors can determine important differences among canopy positions. For example, leaves halfway up the crown, but occupying different orientations, may be subject to marked differences in temperature and water status, depending on the timing of light exposure during the day, as a result of the movements of the sun. Thus, during a summer day, the leaves located on the east side of the canopy receive illumination during the morning, while those on the west side receive full light during the afternoon, when air temperatures tend to be higher. Therefore, it can be expected that leaves facing west are subjected to higher maximum temperatures and lower minimum water potentials. This intracanopy variation should allow the identification of plasticity responses to water stress within a single tree. Despite this, there are almost no studies that have analyzed the phenotypic plasticity associated with the microclimatic differences between the orientations of the canopy due to the movements of the sun [14, 15].

In this paper, we have investigated differences in the characteristics of leaves occupying different orientations of the canopy of isolated specimens of three *Quercus* species under conditions of a Mediterranean climate. High temperatures and summer water deficits are considered the main limiting factors for the growth and survival of tree species growing under these conditions [16]. As explained above, these factors are expected to change between east and west orientations. We selected the morphological and anatomical leaf traits more traditionally related to the regulation of the use of water (including leaf size, dry mass per unit area and tissue density, thickness of the leaf lamina and that of the different leaf tissues, stomatal length and density, density of trichomes and venation patterns). Indeed, a smaller leaf size [17], higher major vein density [18], greater thickness and mass per unit of leaf area [4] and greater density of stomata [19] and trichomes per unit area [20] have been commonly interpreted as mechanisms that allow increasing leaf resistance against drought, since these traits are frequently associated with conditions of greater water stress.

Because the leaves of different orientations in the canopy are most likely subjected to different temperatures and water status throughout the day, we postulated the existence of differences in these foliar traits between eastern and western orientations. It was expected that the leaves within the west orientation of the canopy would exhibit features of drought resistance, such as a smaller area and a higher vein density, greater mass per unit area and thickness, greater pubescence and higher stomatal density, while the opposite would occur in leaves occupying the east orientation. To our knowledge, only two studies have described changes in leaf characteristics in response to microclimatic changes between west and east canopy orientations. The first study [14] was limited to a single evergreen species (olive tree), and, in the other one, two evergreen trees (holm oak and cork oak) were analyzed [15]. Neither of these studies compared possible differences in plasticity among canopy positions in species with different leaf habits (deciduous and evergreen). Additionally, neither study included the analysis of possible differences in traits, such as the density of trichomes and stomata, stomatal pore size or venation patterns, based on canopy orientation. In this study, we have also analyzed differences in chlorophyll and soluble protein content to test the hypothesis that the allocation of photosynthetic resources should be greater in the leaves of the east orientation, which are supposed to photosynthesize under more favorable conditions throughout the day. The main objectives of this work were: 1) to verify if the leaf traits traditionally associated with tolerance to drought actually respond to differences in the intensity of water stress between canopy orientations in the absence of differences in other environmental factors and genotype; and 2) to check if the phenotypic plasticity exhibited in the leaf traits varying between the two orientations is of the same intensity in the three different species. This information would help us to provide a basis for understanding the responses of these species to climate change.

## Materials and methods

### Study species and area

Three species were included in the study: the deciduous *Quercus faginea* Lam. with an average leaf duration of approximately seven months, and the evergreen species *Quercus suber* L. and *Quercus ilex L*. *subsp ballota (Desf*.*) Samp*., with a leaf longevity of approximately 14 and 24 months respectively [21]. All three are typical Mediterranean tree species occupying large areas of open woodlands in the interior of the Iberian Peninsula, and were selected from a site close to the city of Salamanca (central-western Spain) with coordinates 41°08´49.02”N, 5°47´17.38”W. The altitude of this area is approximately 830 m above sea level and the climate is cold Mediterranean. The mean annual temperature ranges from 11–13°C and the mean temperature in the coldest month is between 2 and 6°C and between 20 and 24°C in the hottest month (National Institute of Meteorology, Valladolid Centre). The mean annual rainfall ranges from 500 to 600 mm, with a summer drought period that occurs every year. The soils, dystric Cambisols, are poor in organic matter and nutrients, with a low pH and medium/low water retention capacity [22]. The site consisted of sparse populations (about 50 specimens ha^-1^) of isolated mature trees, over 100 years old, with open pastures between them. The sampling was performed in private properties used for grazing and hunting, with the permission of the land owners. The field studies did not involve any endangered or protected species. For the study we selected five adult individuals per species, with a trunk diameter (taken at 1.3 m height) ranging from 40 to 60 cm and a height of 8–10 m. It was ensured that all the individuals selected for the study were far enough away from neighboring trees so as to not be shaded by them.

### Environmental measurements

Leaf temperature and photosynthetic photon flux density (PPFD) were determined in current-year leaves using a MINI-PAM (Walz GmbH, Germany). The data were collected at four sampling occasions during July of 2017 and 2018, which corresponded to the period of the highest water and thermal stress of each year. Care was taken to select days with stable atmospheric conditions to ensure a fully cloud-free sky throughout the day and temperatures that were only dependent on light intensity. The measurements were taken at approximately 2-h intervals, equally spaced around midday, between sunrise and sunset at two canopy locations (east and west) at mid-height at the periphery of the crown. In total, for each time interval, between 8 and 12 leaves were measured on each side of the canopy and each tree. During the measurement, the leaves were kept in their original positions in order to avoid changes in exposure to solar rays. Two twigs were taken from the same five individual trees and at the same time intervals to measure leaf water potential by means of a pressure chamber (PMS Instruments Co., Mod.1002, Corvallis, OR, US). Water potential measurements were performed on five sampling occasions during July of 2017 and 2018, giving a total of 10 twigs measured on each time interval per side and individual. On each date, the average of the values recorded for the leaves or twigs sampled was calculated for each time interval and for each tree and orientation. In turn, for each tree and orientation, the data corresponding to the same time interval were averaged for all sampling dates.

### Leaf morphological and anatomical measurements

The same five specimens from each species used in the leaf temperature and water potential measurements were selected for morphological and anatomical measurements. Several branches with leaves from east and west crown positions were taken from each canopy at mid-height at the periphery of the crown. The sampling was carried out during the last date on which leaf temperature and water potential were estimated in 2017. In the evergreen species the branches were separated into annual segments (shoots) of different age classes. One single flush of leaf growth was observed in all species. Only the leaves produced during the current year were included in the study. The list of traits analyzed is reported in Table 1.

**Table 1:** Summarizing table of the measured traits.

Abbreviation	Definition	Units
LA	Leaf area	cm^2^
LMA	Leaf mass per unit area	g m^-2^
LD	Leaf density	mg cm^-3^
LT	Total thickness of leaf lamina	μm
E_AD_	Adaxial epidermis thickness	μm
E_AB_	Abaxial epidermis thickness	μm
PT	Palisade tissue thickness	μm
ST	Spongy tissue thickness	μm
SD	Stomatal density	no. mm^-2^
SPL	Stomatal pore length	μm
TD	Trichome density	no. mm^-2^
MaVD	Major vein density	mm mm^-2^
MiVD	Minor vein density	mm mm^-2^
TVD	Total vein density	mm mm^-2^
CF _area_	Chlorophyll content per unit leaf area	g m^-2^
CF _mass_	Chlorophyll content per unit leaf mass	mg g^-1^
PR _area_	Soluble protein content per unit leaf area	g m^-2^
PR _mass_	Soluble protein content per unit leaf mass	mg g^-1^

The morphological traits were analyzed in 50 leaves randomly selected from each species and crown orientation (10 leaves per tree at each side of the crown). Total projected leaf area (LA, cm^2^) was determined using image analysis (Delta-T Devices LTD, Cambridge, UK). The samples were oven-dried at 70°C to constant mass and the total dry mass per leaf was determined. From the data obtained, we calculated the leaf mass per area (LMA, g m^-2^) and tissue density (LD, mg cm^-3^) (dry mass/volume). The data of the 10 leaves selected in each case were averaged to obtain a value per tree and orientation.

For the anatomical analysis, 5 x 5 mm pieces were removed from the middle part of two leaves, taken from each tree and orientation, with a razor blade and fixed in formaldehyde-acetic acid (FAA; 35–40% formaldehyde, 70% ethanol and 100% acetic acid, 1:8:1 v/v) for at least 24 h. The material was then dehydrated in an ethanol series and embedded in methacrylate liquid for 72 hours. Transverse leaf sections (5–6 μm thick) made on a HM 350S Rotary Microtome (Microm International GmbH, Germany) were mounted on glass slides. Images of each section were obtained using a digital camera (Nikon Sight DS-smc, Nikon Instruments INC, USA) mounted on a microscope (Nikon Eclipse 90i, Nikon Instruments INC, USA). Total leaf thickness (LT, μm), palisade (PT, μm) and spongy mesophyll thickness (ST, μm) and adaxial (E_AD_, μm) and abaxial (E_AB_, μm) epidermal thickness were calculated using the software ImageJ (http://rsb.info.nih.gov/ij/; [23]). Two measurements of the total thickness of the leaf and each tissue were taken from each photograph, in all cases at the midpoint between two vascular bundles.

Quantitative measurements of leaf stomata and trichomes were made by analyzing images of another subsample of leaves using a scanning electron microscope (SEM EVO HD25, Carl Zeiss Microscopy, Germany). Only the abaxial epidermis was analyzed, since no stomata were found on the adaxial side of the leaves of the three species [24–26]. The abaxial side of two leaves, taken from each tree and orientation, was shaved using a razor blade and sectioned into 0.5 cm^2^ vein-free portions that were then fixed in glutaraldehyde 2.5% in 0.1M phosphate buffer and pH 7.4. The samples were transferred to the Microscopy Service of the University of Salamanca where, after washing, dehydration and metallization, photographs were taken in two fields for each leaf fraction. The leaf stomatal density was expressed as the number of stomata per unit leaf area (SD, number mm^-2^). Stomatal pore length (SPL, μm) was defined as the length in micrometers between the junctions of the guard cells at each end of the stomata [27]. The number of trichomes was counted as the total number of trichome branches, with each branch of stellate trichomes counted as a trichome [28], and expressed per unit of leaf area (TD, number mm^-2^).

For measuring the veins, the four leaves selected per tree and orientation were fixed for 5 hours in FAA (formalin:acetic acid: 50% ethanol, 5:5:90) to avoid the tissues from becoming distorted and rinsed in 50% ethanol and distilled water. The samples were subsequently cleared with 2M NaOH for 5 hours and washed consecutively with a bleach solution and 50% and 70% ethanol (2 hours each). For visualization of vascular tissues, the leaves were stained with a 0.01% (w/v) safranine solution in 70% ethanol for 3 min and extensively rinsed in water. Images were immediately acquired with a Leica M205 FA stereo microscope equipped with a Leica DFC495 camera (Leica Microsystems, Germany) and the lengths and diameters of first-, second- and third-order veins (“major veins”) and those of the remainder veins (“minor veins”) were measured using the ImageJ software [23]. We measured total length of the mid-vein (first order). In addition, the density of the remainder vein orders was averaged for three subsampled regions, taken centrally in the top, middle, and bottom third of the leaf. For each vein order, the density was calculated as the length divided by leaf area. The major vein density (MaVD) was then obtained as the sum of the first-, second- and third-order vein densities. The remainder veins were used to estimate minor vein density (MiVD).

### Leaf chemical measurements

For the chemical analyses, a disk (0.28 cm^2^) was cut from 15 intact current-year leaves, from each orientation, taken from each of the five specimens selected for each species. At the laboratory, plant material was weighed and immediately placed into liquid nitrogen and kept at -80°C until further analysis. The Agrisera method (Sweden) was used for carrying out the protein extraction and for determining the chlorophyll and protein content. Chlorophyll was measured according to Wathley & Arnon (1963) [29] and the total soluble protein was measured according to Bradford (1976) [30]. The dry mass and LMA of the leaves used for the analyses were also determined and the chlorophyll (CF) and protein (PR) contents of leaves were expressed per unit dry mass and per unit leaf area.

### Data analysis

Data were analyzed for all of the species together through a repeated-measures ANOVA, blocked for given trees, with the species and canopy locations as factors [10]. Next, for each species, data were analyzed using a one-way, repeated-measures ANOVA, blocked by tree, for the effects of canopy location [10]. The Levene test was used to check for homogeneity of variance. For the traits in which significant differences were detected between orientations, a plasticity index (PI) ranging from 0 to 1 was calculated as (maximum value-minimum value)/maximum value [31, 32]. This index permits plasticity comparisons among traits recorded in different units. The SPSS statistical package was used to analyze the data (SPSS Inc., Chicago, IL).

## Results

### Differences in environmental conditions between canopy positions

**Fig 1:**
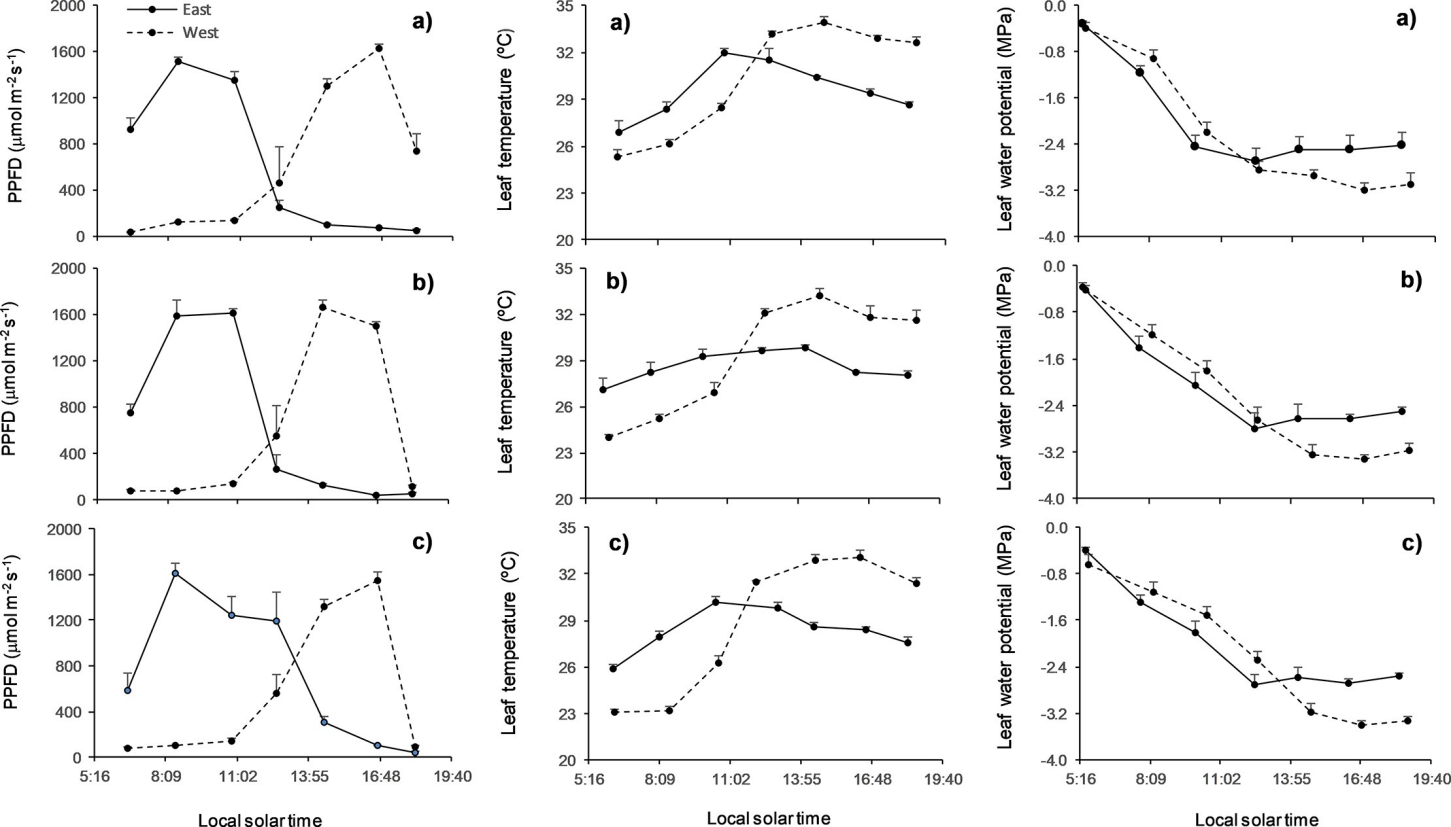
Diurnal changes in photosynthetic photon flux density (PPFD) incident on leaf surfaces, leaf temperature and leaf water potential for the two canopy orientations in *Q*. *faginea* (a), *Q*. *suber* (b) and *Q*. *ilex* (c). Data are represented as mean + SE (n = five trees).

Diurnal variations in PPFD were different for the two canopy locations (Fig 1). On the eastern side of the canopy, PPFD reached maximum values early in the morning and decreased after midday due to self-shading by the crown. On the western side, PPFD levels reached maximum values during the afternoon. Despite these differences in temporal distribution of irradiance levels, there were no significant differences between the two sides of the crown in the average PPFD intercepted or in maximal PPFD values according to one-way repeated measures ANOVA.

The leaf temperature changed throughout the day with clear differences between orientations. Leaves facing east tended to have higher temperatures than those on the western side during the morning, reaching maximum values around midday, and showing slight decreases throughout the afternoon (Fig 1). However, on the western side, leaf temperatures increased steadily during the morning and part of the afternoon, reaching maximum values at around 14:00 h (local solar time), and without showing decreases until sunset. In this way, the leaves of the western orientation reached maximum daily temperatures around 10% higher than those of the eastern orientation, resulting in significant differences in all species (P = 0.01, P = 0.002 and P = 0.00034, significance levels for differences between east and west orientation in *Q*. *faginea*, *Q*. *suber* and *Q*. *ilex* respectively; one-way repeated measures ANOVA).

Maximum leaf water potential was recorded shortly before dawn in the two orientations, without significant differences between them. Subsequently, leaf water potential decreased sharply with the increase in temperature during the day (Fig 1). In the eastern orientation, the minimum daily water potential was recorded earlier than for the western side. In contrast, decreases in leaf water potentials were prolonged until later in the day for the western orientation, and the afternoon recovery was less pronounced. Thus, mean minimum leaf water potentials differed significantly between orientations, with values always lower in leaves on the west side than on the east (P <0.0001, significance levels for differences between orientations in *Q*. *faginea*, *Q*. *suber* and *Q*. *ilex*). The differences in the minimum daily water potential between orientations were somewhat more pronounced than for leaf temperature, but also of similar magnitude in the three species studied (average values around 20% lower in the west compared to the east).

### Differences among canopy positions in leaf traits

Leaf size differed between orientations, with lower values on the west side of the crown in all species (Table 2), although the differences were only marginally significant for *Q*. *faginea*. Contrary to expectations, there were no differences between canopy orientations in LMA, total thickness or density. However, although no difference in the total thickness was obtained in any case, differences were observed in the proportion of the two types of parenchyma. Thus, in the three species, the leaves facing west showed a significantly higher thickness of palisade parenchyma than those in the east, while the opposite occurred for the thickness of the spongy parenchyma, although the differences were not significant for *Q*. *suber* (Table 2). The leaves on the west also showed a number of stomata and trichomes per unit area higher than those of the east, although without differences in the stomatal pore length (Table 3). Finally, no difference was obtained between orientations in vein density (Table 3).

**Table 2:** Average leaf morphological traits at two canopy orientations (S.E. in parentheses, n = 5 trees) for three tree species. Results of analyses of variance for individual species in tests for effect of canopy position (CP) and for all species together in tests for species differences (SP), effect of canopy position (CP) and species x canopy position (SP x CP).

			CP effect	For all species together, effect (P) of		
Species	East	West	(P)	SP	CP	SP x CP
	**LA (cm**^**2**^**)**			< 0.001	< 0.001	ns
*Q*. *faginea*	9.04 (0.43)	6.90 (0.42)	0.062			
*Q*. *suber*	7.39 (0.28)	5.51 (0.47)	0.001			
*Q*. *ilex*	3.57 (0.33)	2.37 (0.16)	0.004			
	**LMA (g m**^**-2**^**)**			< 0.001	ns	ns
*Q*. *faginea*	149 (6.15)	144 (7.51)	ns			
*Q*. *suber*	185 (5.47)	190 (1.70)	ns			
*Q*. *ilex*	237 (4.04)	236 (3.04)	ns			
	**LD (mg cm**^**-3**^**)**			ns	ns	ns
*Q*. *faginea*	771 (36.7)	724 (36.7)	ns			
*Q*. *suber*	697 (34.7)	715 (20.5)	ns			
*Q*. *ilex*	690 (11.9)	697 (27.2)	ns			
	**LT (μm)**			< 0.001	ns	ns
*Q*. *faginea*	199 (5.60)	196 (5.99)	ns			
*Q*. *suber*	267 (9.12)	266 (5.31)	ns			
*Q*. *ilex*	349 (5.76)	343 (11.5)	ns			
	**E**_**AD**_ **(μm)**			< 0.001	ns	ns
*Q*. *faginea*	23 (1.57)	22 (0.70)	ns			
*Q*. *suber*	15 (1.04)	14 (0.49)	ns			
*Q*. *ilex*	29 (2.99)	28 (2.38)	ns			
	**E**_**AB**_ **(μm)**			0.058	ns	ns
*Q*. *faginea*	10 (0.86)	10 (0.52)	ns			
*Q*. *suber*	9 (0.87)	8 (0.63)	ns			
*Q*. *ilex*	10 (0.64)	10 (0.62)	ns			
	**PT (μm)**			< 0.001	< 0.001	ns
*Q*. *faginea*	92 (1.74)	98 (1.36)	0.050			
*Q*. *suber*	160 (4.93)	171 (1.23)	0.020			
*Q*. *ilex*	175 (1.57)	189 (2.98)	0.040			
	**ST (μm)**			< 0.001	< 0.001	ns
*Q*. *faginea*	73 (3.36)	65 (3.20)	0.040			
*Q*. *suber*	82 (3.60)	72 (4.27)	ns			
*Q*. *ilex*	136 (4.76)	115 (6.43)	0.011			

**Table 3:** Average stomatal and venation traits and trichome density at two canopy orientations (S.E. in parentheses, n = 5 trees) for three tree species. Results of analyses of variance for individual species in tests for effect of canopy position (CP) and for all species together in tests for species differences (SP), effect of canopy position (CP) and species x canopy position (SP x CP).

			CP effect	For all species together, effect (P) of		
Species	East	West	(P)	SP	CP	SP x CP
	**SD (no. mm**^**-2**^**)**			< 0.001	0.003	ns
*Q*. *faginea*	524 (11.9)	576 (12.7)	0.020			
*Q*. *suber*	527 (18.9)	594 (21.1)	0.050			
*Q*. *ilex*	403 (27.2)	501 (25.2)	0.001			
	**SPL (μm)**			0.035	ns	ns
*Q*. *faginea*	12.0 (0.53)	11.4 (0.38)	ns			
*Q*. *suber*	10.1 (0.23)	10.6 (0.47)	ns			
*Q*. *ilex*	11.7 (0.56)	11.9 (0.26)	ns			
	**TD (no. mm**^**-2**^**)**			< 0.001	< 0.001	ns
*Q*. *faginea*	145 (8.44)	177 (3.85)	0.008			
*Q*. *suber*	288 (4.46)	330 (15.1)	0.061			
*Q*. *ilex*	151 (8.07)	194 (5.39)	0.001			
	**MaVD (mm mm**^**-2**^**)**			0.013	ns	ns
*Q*. *faginea*	1.63 (0.08)	1.62 (0.01)	ns			
*Q*. *suber*	1.59 (0.04)	1.64 (0.11)	ns			
*Q*. *ilex*	1.94 (0.12)	1.85 (0.04)	ns			
	**MiVD (mm mm**^**-2**^**)**			< 0.001	ns	ns
*Q*. *faginea*	14.7 (0.95)	14.5 (0.96)	ns			
*Q*. *suber*	8.63 (0.41)	8.31 (0.37)	ns			
*Q*. *ilex*	10.4 (0.86)	10.4 (0.53)	ns			
	**TVD (mm mm**^**-2**^**)**			< 0.001	ns	ns
*Q*. *faginea*	16.3 (1.02)	16.2 (0.97)	ns			
*Q*. *suber*	10.2 (0.42)	9.95 (0.41)	ns			
*Q*. *ilex*	12.4 (0.98)	12.2 (0.57)	ns			

**Fig 2:**
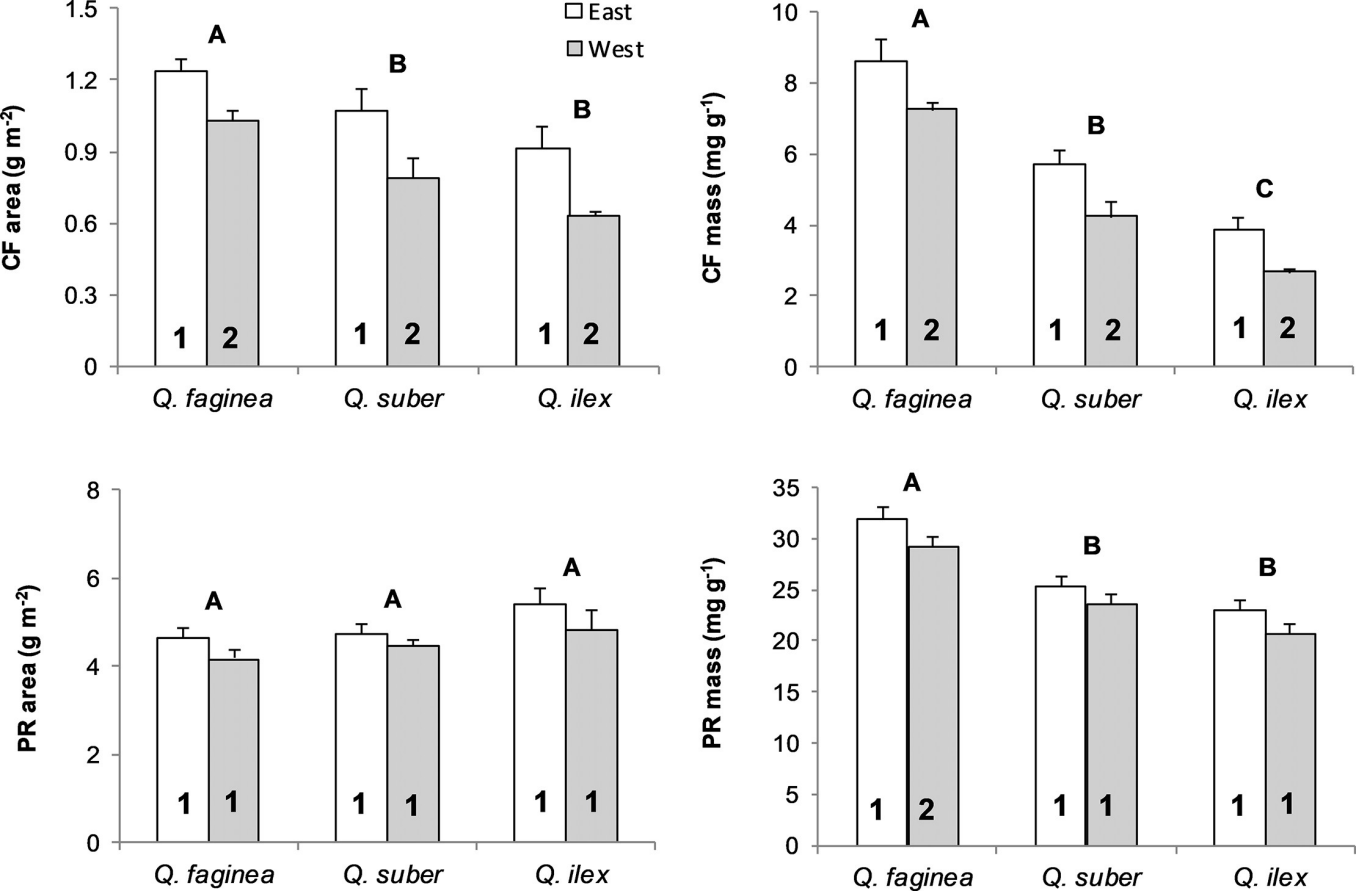
Mean + SE (n = five trees) leaf chlorophyll (CF) and soluble protein (PR) content at two canopy positions. Different numbers within bars indicate significant differences (P < 0.05) between canopy positions. Different letters above bars indicate significant differences (P < 0.05) between species pairs (Posthoc Tukey HSD test).

Total soluble protein content did not differ between the leaves facing east and west, with the exception of *Q*. *faginea*, which showed higher protein concentration per unit leaf mass in the eastern orientation. However, the chlorophyll content was significantly higher in the leaves in the eastern position in all species, expressed as both per unit leaf area and per unit leaf mass (Fig 2).

### Plasticity index among canopy positions for the different traits and species

The interaction term (species x orientation) was not significant for any of the traits analyzed (Tables 2 and 3). This result, in principle, would suggest that the intensity of the differences between the leaves of the two orientations did not differ enough between the three species to be significant. Even so, the plasticity index for the traits that did vary significantly between orientations tended to increase with leaf longevity of the different species. In all cases, the species with the highest leaf longevity had the highest PI values (Table 4). This indicates that, in response to an east-west change in maximum leaf temperature and minimum water potential of similar magnitude, *Q*. *ilex* was the species that showed the most marked differences between leaves occupying different positions of the crown. In the three species, leaf size was the trait that varied most significantly between orientations, followed by the chlorophyll content, while the less intense changes were observed for parenchyma thickness (Table 4).

**Table 4:** Leaf plasticity indices [(maximum value-minimum value)/maximum value] for the leaf traits showing significant differences between east and west canopy orientations. Data are means (SE in parentheses) from five different trees.

	LA (cm^2^)	PT (μm)	ST (μm)	SD (no. mm^-2^)	TD (no. mm^-2^)	CFmass (mg g^-1^)	CFarea (g m^-2^)
*Q*. *faginea*	0.266 (0.051)	0.064 (0.022)	0.147 (0.028)	0.090 (0.022)	0.182 (0.038)	0.153 (0.043)	0.163 (0.043)
*Q*. *suber*	0.260 (0.039)	0.067 (0.018)	0.141 (0.060)	0.127 (0.023)	0.127 (0.039)	0.256 (0.076)	0.246 (0.085)
*Q*.*ilex*	0.328 (0.028)	0.072 (0.022)	0.155 (0.032)	0.196 (0.026)	0.223 (0.029)	0.282 (0.067)	0.284 (0.068)

## Discussion

Significant plasticity was observed within the crown in response to differences in environmental conditions between east and west orientations. Several of the leaf traits analyzed in the present study have traditionally been associated with drought tolerance. As a consequence, differences in these traits through environmental gradients have been understood as responses to changes in the intensity of water stress. In principle, the environmental differences between east and west orientations should be exclusively derived from the changes in the position of the sun throughout the day, since maximum radiation levels were similar for both sides of the crown. Despite this, since the maximum levels of radiation at the western side coincided with the time of the day with highest temperatures, there were clear differences between both orientations in the intensity of maximum water stress experienced throughout the day. The leaves of the west orientation always showed higher daily maximum temperatures and lower minimum water potentials than those of the eastern orientation. This suggests that the leaf traits exhibiting significant differences between orientations responded mainly to the intensification of thermal and hydric stress across the east-west gradient, which would confirm the role of such traits in heat and water stress tolerance and the control of water loss by transpiration. In the present study the response of leaf traits to changes in orientation was not general, but limited to leaf size, density of stomata and trichomes, the percentage of spongy and palisade tissues and chlorophyll content, these being the only traits that varied between orientations in the three species studied.

Leaf size was the trait exhibiting the most pronounced differences between orientations (between 20 and 30% lower individual mean leaf area in the western position with respect to the east orientation). Smaller leaf size is a trait typical of drier sites that has been interpreted as a mechanism that reduces the thickness of the boundary layer, which increases heat loss by convection and results in leaf temperatures closer to air temperature and lower rates of transpiration in smaller than in larger leaves [17]. In addition to their smaller size, the leaves facing west also showed a significantly higher density of stomata than those of the east. Stomata are known to play an important role in the control of transpiration and leaf temperature, in addition to the diffusion of CO_2_ [33], and larger stomatal density has been frequently found associated with increased water stress [19, 34]. According to some authors, this increase in the number of stomata is accompanied by a decrease in pore size, which would allow a faster opening speed and higher water use efficiency under favorable conditions [35, 36]. However, in our case, the plasticity between crown orientations was limited to the number of stomata per unit area, with no differences in pore size, which would corroborate the reduced plasticity observed in stomatal lengths compared to stomatal density as reported by other authors [10, 37].

The higher maximum temperatures and the lower minimum water potentials of the leaves occupying the west position indicate a greater risk of photoinhibition and larger potential rates of transpiration. Therefore, it would be expected that these leaves should have higher pubescence than those of the east in order to prevent both risks. The role of trichomes has been related to the control of transpirational water loss, contributing to reducing leaf temperature and increasing the thickness of the boundary layer [38]. Although the latter effect could negatively influence heat exchange, when the trichomes are concentrated around stomatal pores, it may have a stronger effect on the restriction of water vapor exchange. The presence of trichomes also has protective functions against excessive radiation, reducing susceptibility to photoinhibition [39]. Actually, the trichomes were present only on the abaxial surface of the leaves. Given that all of our species are hypostomatous [24–26], the presence of trichomes seems to be especially associated with the need to restrict water loss by transpiration on the western side, rather than protecting leaf surfaces from excess radiation. Some authors have proposed that the increase in the density of trichomes could also reduce the diffusion of CO_2_ into the stomatal cavity and thus photosynthetic activity [40, 41]. However, the larger abundance of stomata in the leaves on the west could allow for a rapid increase in stomatal conductance, maximizing the diffusion of CO_2_ for photosynthesis under favorable environmental conditions [33, 36] and thus compensating the unfavorable effects of pubescence on the exchange of CO_2_.

We did not find, however, any response to the differences in the intensity of water stress between canopy orientations in the case of leaf thickness or LMA. This is, without doubt, the most striking result of our study, because a larger LMA is one of the leaf traits most frequently associated with the increase in water stress across different environments. The absence of responses in LMA also contrasts with the strong plastic responses of this trait to the irradiance gradient within the canopy seen by other authors [9]. It has been postulated that a leaf with a large LMA is better able to store water and maintain more stable hydraulic functioning during droughty periods, which could explain why leaf thickness and LMA tend to increase with site aridity [3, 42, 43]. Among the different leaf traits, LMA has attracted the most attention, mainly because changes in LMA are accompanied by changes in other characteristics, such as leaf life span and fiber and nutrient contents [44], which lead to important trade-offs between productivity and persistence [45]. A greater LMA has been traditionally interpreted as a trait aimed at guaranteeing leaf survival, acting as protection against different environmental factors such as attack by herbivores, low winter temperatures and drought [46–48]. However, according to the results of our study, leaves on the western side of the crown responded to drought stress mainly by producing smaller leaves with higher density of stomata and trichomes, without incurring costs due to an increased LMA or total thickness. In principle, one might argue that the traits we expected to respond to the change in orientation require stronger environmental contrasts in order to induce a response. However, the environmental differences between east and west were intense enough to trigger significant responses in other traits. We would expect LMA to respond in a similar manner if this trait were as important for stress tolerance as the other traits. The changes in LMA depend on changes in density and/or thickness [49]. According to some researchers, the increase in thickness would be a typical response to an increase in radiation, whereas decreases in water availability would lead to increases in leaf tissue density [47]. If there are no differences in the levels of total radiation received by the leaves of the two crown orientations, this would explain the absence of differences in thickness of the leaves between east and west. Despite the differences in the minimum water potentials, we did not find a difference in density or LMA. However, we found significant differences in the thickness of the two kinds of mesophyll tissues, although the changes in these traits were much less marked than for the other traits. In the three species, the west-facing leaves showed a greater palisade tissue thickness, whereas the spongy tissue layer was thicker in the leaves in the eastern orientation. In principle, given the differences in the proportion of spongy and palisade parenchyma, we could expect to find differences in density between both orientations, but, apparently, the small difference in the proportion of both tissues was compensated by other factors, which resulted in similar densities for both sides of the crown. Perhaps, if there are higher percentages of palisade mesophyll under drier and hotter conditions, a stronger gradient in water availability in different environments would result in differences in density, as other authors have reported. In fact, although this has been less studied, changes in the thickness of different types of parenchyma have also been associated with differences in water availability in other studies [50, 51]. A thicker palisade tissue could be an indication of higher photosynthetic capacity [52]. Therefore, a thicker palisade tissue would allow the west-facing leaves to maximize photosynthesis when conditions are favorable throughout the day and growing season. In addition, a higher amount of photosynthetic machinery per unit area would contribute to increasing water use efficiency [53], because the transpiration rate would be independent from the amount of photosynthetic machinery per unit area [54]. Positive correlations between palisade thickness and leaf hydraulic conductance have also been proposed by some authors [55]. Since the palisade tissue is rather hydraulically isolated from the bulk of the transpiration stream [56], this could allow an increasing water use efficiency that compensates for the most unfavorable water conditions on the western side.

In addition, no differences in vein density were found between the crown orientations in any of the species and, therefore, no relationship between leaf vein density and leaf size was detected. We expected the west-facing leaves would be smaller in size and exhibit larger vein density compared to those in the east. The inverse relationship between vein density and leaf size has been confirmed in numerous interspecific studies [34, 57, 58]. According to some authors [18], the drought tolerance and protection conferred by higher vein density in smaller leaves would justify the greater abundance of smaller leaves in drier and more exposed habitats, since a high vein density has been frequently associated with larger leaf hydraulic conductance and gas exchange rates and greater resistance of the leaf to hydraulic conductivity losses [18]. However, the relationship between leaf size and vein density is not clear at the intraspecific level. Uhl and Mosbrugger (1999) [59], for example, observed that there was no correlation with leaf size in *Quercus petraea* leaves, although vein densities increased with the decrease in leaf size in *Acer monspessulanum*. Some authors have also pointed out that leaf venation patterns in many species would be conservative and, therefore, much less sensitive to changes in environmental conditions than other traits [60, 61], which also fits our results.

Finally, no significant variation was found in the total soluble protein content between the two canopy orientations, although in *Q*. *faginea* the protein concentration per unit mass was significantly higher at the east side. Although leaf N content was not analyzed in this work, Escudero et al. (2013) [15] did not find differences in N concentrations between the leaves of the east and the west sides. Nitrogen varies with light availability in the plant crown for optimizing daily crown photosynthesis [62]. The absence of differences in protein content per unit leaf area between orientations could reflect the similar levels of total radiation received throughout the day in both orientations. By contrast, the concentrations of chlorophyll were significantly lower in the west. Given the absence of differences in N and protein contents, the loss of chlorophyll in the leaves of the west may be a direct effect of excess radiation at the time of the day with highest leaf temperatures and lowest water potential rather than the consequence of differences in resource allocation between both sides of the crown. Reducing photodamage to the photosynthetic system under excess radiation is one of the roles attributed to the lower total chlorophyll content that frequently accompanies conditions of greater water stress [63]. Metabolic impairment of photosynthesis tends to occur when maximum daily stomatal conductance drops below a given level [64]. Given the differences in water potential and maximum leaf temperatures between both sides of the crown, leaves facing west should have a larger probability of reaching this critical stomatal conductance, which would lead to a reduction in chlorophyll contents.

Assuming that the only environmental differences between the east and west sides of the canopy are maximum leaf temperatures and minimum water potentials, the analysis carried out in this paper suggests that the leaf traits that exhibited changes between both sides are those more strictly linked to heat and drought tolerance. Contrary to our expectations, neither LMA nor vein density exhibited such differences. This would suggest that both traits do not play such an important role as previously suspected, although obviously more detailed analyses are required to confirm this conclusion. It is then possible that the changes observed in LMA along climatic gradients are responses to other environmental factors rather than to gradients in water stress. In fact, the relationships between climate and leaf traits published until now tend to show a low explained variance [47]. The most probable explanation is that across climatic gradients many other plant adaptive characteristics besides leaf traits may contribute to obscure the role of leaf traits in determining the competitive ability of plant species along climatic gradients [47]. Similarly, the capacity of most plant traits for predicting demographic performance is weak, particularly in tree communities [65]. Comparisons made within an individual plant allow minimizing the variation in other traits and this may help to clarify the relationships between leaf traits and stress tolerance.

*Q*. *ilex* was the species that exhibited the greatest plasticity for the traits showing differences between positions, whereas the deciduous *Q*. *faginea* showed minimum PI values for most leaf traits. This result contradicts the finding of other studies which found greater plasticity in deciduous seedlings compared to evergreen [31], or similar levels of plasticity in adult specimens of species of both habits [10] in response to variations in radiation levels. However, the greater plasticity observed in this study in species with longer-living leaves is consistent with the fact that these species exhibit more dramatic changes in leaf traits (i.e. larger plasticity) along ontogeny [66, 67]. Since the probability of stress events increases with increasing foliage longevity [68], the traits that confer tolerance to environmental stress become increasingly important with increasing leaf life span. In this sense it seems logical that the differences in the leaf traits between canopy positions with different stress levels, are also greater in leaves with a longer life expectancy. This provides additional proof that the observed changes in leaf traits between the eastern and western sides of the canopy are in response to more severe drought condition suffered on the west-facing side of the canopy.

## Concluding remarks

Investigating intracanopy plasticity may help clarify the role of different leaf traits as responses to specific environmental factors, because of the limited environmental variation among canopy positions in comparison with larger-scale gradients. In the present paper, we have identified a significant gradient between east and west canopy orientations in isolated trees that had been rarely investigated until now. Our approach allowed investigating specific responses of leaf traits to heat and drought stress in absence of other confounding variables. The plastic responses of the leaves to the east-west gradient may be decisive to improve leaf performance in each side of the crown in a water-limited environment, thus contributing to the optimization of carbon assimilation and water relations at the level of the entire canopy.

## Supporting information

S1 Table Average leaf characteristics for the five trees sampled. (PDF)
