# Supplementary material for: Foliar plasticity related to gradients of heat and drought stress across crown orientations in three Mediterranean Quercus species
Source: PLoS One. 2019 Oct 28;14(10):e0224462. doi: 10.1371/journal.pone.0224462 (PMC6816560; doi:10.1371/journal.pone.0224462)
Supplement: S1 Table — (PDF) [file pone.0224462.s001.pdf]

SPECIES	Crown orientation	Maximum daily PPFD (μmol m^{-2} s^{-1})	Maximum daily leaf temperature (ºC)	Minimum daily leaf water potential (MPa)	Leaf area (cm^2)	LMA (g m^{-2})	Leaf density (mg cm^{-3})
Q. faginea	EAST	1454	32.4	-2.67	9.28	170	811
Q. faginea	EAST	1494	30.6	-2.64	9.42	137	665
Q. faginea	EAST	1433	32.3	-2.71	7.44	154	865
Q. faginea	EAST	1569	31.3	-2.75	10.03	144	807
Q. faginea	EAST	1625	31.7	-2.81	9.01	139	708
Q. faginea	WEST	1713	33.2	-3.16	6.39	164	851
Q. faginea	WEST	1581	34.4	-3.20	5.99	122	631
Q. faginea	WEST	1582	34.1	-3.35	8.34	156	726
Q. faginea	WEST	1727	34.1	-3.33	6.41	136	679
Q. faginea	WEST	1510	34.2	-3.33	7.35	141	730
Q. suber	EAST	1709	29.1	-2.68	6.95	187	742
Q. suber	EAST	1572	31.2	-2.70	7.51	195	785
Q. suber	EAST	1538	28.5	-2.74	7.56	196	725
Q. suber	EAST	1661	29.3	-2.89	8.27	180	601
Q. suber	EAST	1575	28.0	-3.06	6.67	166	633
Q. suber	WEST	1832	33.8	-3.20	4.66	185	662
Q. suber	WEST	1758	33.6	-3.23	5.74	191	718
Q. suber	WEST	1591	34.3	-3.38	6.31	187	678
Q. suber	WEST	1430	32.8	-3.34	6.65	194	757
Q. suber	WEST	1665	32.8	-3.65	4.18	193	763
Q. ilex	EAST	1379	29.5	-2.58	3.73	247	678
Q. ilex	EAST	1899	30.6	-2.60	3.01	234	685
Q. ilex	EAST	1693	30.2	-2.70	3.32	235	715
Q. ilex	EAST	1518	29.6	-2.82	3.01	245	717
Q. ilex	EAST	1542	30.7	-2.92	4.76	225	653
Q. ilex	WEST	1452	32.6	-3.28	2.48	243	765
Q. ilex	WEST	1624	33.9	-3.30	1.92	235	647
Q. ilex	WEST	1461	32.9	-3.47	2.51	228	624
Q. ilex	WEST	1399	33.9	-3.40	2.13	243	707
Q. ilex	WEST	1794	33.5	-3.60	2.84	231	744

SPECIES	Crown orientation	Leaf thickness (μm)	Adaxial epidermis thickness (μm)	Abaxial epidermis thickness (μm)	Palisade tissue thickness (μm)	Spongy tissue thickness (μm)	Stomatal density (number mm^{-2})
Q. faginea	EAST	210	26	13	92	78	547
Q. faginea	EAST	206	25	8	98	73	506
Q. faginea	EAST	178	23	9	88	76	566
Q. faginea	EAST	193	17	11	89	70	517
Q. faginea	EAST	196	25	12	92	68	482
Q. faginea	WEST	193	20	10	96	64	620
Q. faginea	WEST	178	21	10	99	60	542
Q. faginea	WEST	215	23	9	103	76	580
Q. faginea	WEST	200	24	10	96	67	566
Q. faginea	WEST	193	22	12	97	58	572
Q. suber	EAST	252	14	8	156	79	465
Q. suber	EAST	249	17	7	150	76	543
Q. suber	EAST	270	15	8	164	80	557
Q. suber	EAST	300	13	12	173	96	567
Q. suber	EAST	263	18	10	155	81	503
Q. suber	WEST	280	15	9	170	86	568
Q. suber	WEST	266	15	7	170	75	638
Q. suber	WEST	276	15	10	171	75	651
Q. suber	WEST	256	14	9	175	63	543
Q. suber	WEST	253	13	7	170	64	572
Q. ilex	EAST	364	30	10	178	149	465
Q. ilex	EAST	342	32	11	174	137	436
Q. ilex	EAST	328	33	8	170	134	440
Q. ilex	EAST	342	17	12	175	132	345
Q. ilex	EAST	344	32	9	178	128	331
Q. ilex	WEST	317	24	11	187	111	571
Q. ilex	WEST	363	31	11	190	125	544
Q. ilex	WEST	366	32	12	198	120	491
Q. ilex	WEST	344	34	9	187	115	464
Q. ilex	WEST	310	22	9	180	102	434

SPECIES	Crown orientation	Stomatal pore length (μm)	Trichomes density (number mm^{-2})	Major vein density (mm mm^{-2})	Minor vein density (mm mm^{-2})	Total vein density (mm mm^{-2})	Chlorophyll content per unit leaf area (g m^{-2})
Q. faginea	EAST	13.0	159	1.400	11.57	12.97	1.27
Q. faginea	EAST	12.7	138	1.773	15.55	17.32	1.22
Q. faginea	EAST	11.8	169	1.689	16.18	17.87	1.07
Q. faginea	EAST	12.4	140	1.793	16.61	18.40	1.31
Q. faginea	EAST	10.0	120	1.499	13.46	14.96	1.33
Q. faginea	WEST	12.5	187	1.605	13.81	15.42	1.18
Q. faginea	WEST	12.0	165	1.656	17.68	19.34	1.01
Q. faginea	WEST	10.7	183	1.634	14.72	16.36	0.99
Q. faginea	WEST	11.2	179	1.610	14.84	16.45	0.92
Q. faginea	WEST	10.5	173	1.608	11.72	13.33	1.07
Q. suber	EAST	9.7	276	1.744	9.12	10.86	1.20
Q. suber	EAST	10.0	301	1.567	9.23	10.80	1.28
Q. suber	EAST	9.8	283	1.572	9.01	10.58	1.09
Q. suber	EAST	10.0	294	1.485	8.79	10.28	0.77
Q. suber	EAST	11.0	285	1.591	7.01	8.60	1.03
Q. suber	WEST	9.1	317	1.963	9.42	11.39	1.11
Q. suber	WEST	11.8	338	1.652	7.68	9.33	0.68
Q. suber	WEST	11.2	382	1.746	7.46	9.21	0.69
Q. suber	WEST	10.0	290	1.303	8.15	9.45	0.75
Q. suber	WEST	11.0	324	1.529	8.86	10.39	0.73
Q. ilex	EAST	13.8	169	1.855	10.19	12.05	1.13
Q. ilex	EAST	10.8	166	1.863	10.32	12.19	1.16
Q. ilex	EAST	12.0	151	1.768	9.97	11.74	0.75
Q. ilex	EAST	11.0	146	2.433	13.53	15.96	0.77
Q. ilex	EAST	11.0	124	1.764	8.20	9.97	0.75
Q. ilex	WEST	12.6	206	1.756	8.67	10.42	0.67
Q. ilex	WEST	11.8	197	2.024	11.77	13.79	0.61
Q. ilex	WEST	12.0	205	1.826	10.36	12.18	0.62
Q. ilex	WEST	12.0	182	1.824	9.98	11.80	0.58
Q. ilex	WEST	11.0	181	1.818	11.13	12.95	0.66

SPECIES	Crown orientation	Chlorophyll content per unit leaf mass (mg g^{-1})	Soluble protein content per unit leaf area (g m^{-2})	Soluble protein content per unit leaf mass (mg g^{-1})
Q. faginea	EAST	7.61	4.95	29.73
Q. faginea	EAST	9.16	3.93	29.41
Q. faginea	EAST	6.97	5.26	34.29
Q. faginea	EAST	9.69	4.74	34.92
Q. faginea	EAST	9.70	4.31	31.39
Q. faginea	WEST	7.47	4.27	27.08
Q. faginea	WEST	7.27	3.88	27.88
Q. faginea	WEST	6.36	4.75	30.42
Q. faginea	WEST	7.41	3.68	29.77
Q. faginea	WEST	7.59	4.27	30.32
Q. suber	EAST	6.34	4.06	21.38
Q. suber	EAST	6.39	5.39	26.82
Q. suber	EAST	5.99	4.89	26.79
Q. suber	EAST	4.34	4.57	25.92
Q. suber	EAST	5.47	4.78	25.40
Q. suber	WEST	5.97	4.88	26.33
Q. suber	WEST	3.58	4.45	23.30
Q. suber	WEST	3.67	3.98	20.85
Q. suber	WEST	3.91	4.46	23.85
Q. suber	WEST	3.87	4.52	23.39
Q. ilex	EAST	4.58	5.97	24.06
Q. ilex	EAST	4.96	6.02	25.83
Q. ilex	EAST	3.21	6.45	27.59
Q. ilex	EAST	3.12	2.72	11.05
Q. ilex	EAST	3.35	5.84	26.20
Q. ilex	WEST	2.76	4.41	18.16
Q. ilex	WEST	2.58	3.64	15.51
Q. ilex	WEST	2.70	6.01	26.33
Q. ilex	WEST	2.40	4.68	19.22
Q. ilex	WEST	2.87	5.47	23.75
